# A time to heal: microRNA and circadian dynamics in cutaneous wound repair

**DOI:** 10.1042/CS20220011

**Published:** 2022-04-21

**Authors:** Sandra Fawcett, Raida Al Kassas, Iain M Dykes, Alun TL Hughes, Fawaz Ghali, Kehinde Ross

**Affiliations:** 1 School of Pharmacy and Biomolecular Science, Liverpool John Moores University, Liverpool, United Kingdom; 2 Instiute for Health Research, Liverpool John Moores University, Liverpool, United Kingdom; 3 School of Biological and Environmental Sciences, Liverpool John Moores University, Liverpool, United Kingdom; 4 School of Computer Science and Mathematics, Liverpool John Moores University, Liverpool, United Kingdom

**Keywords:** circadian clock, exosomes, keratinocytes, microRNA, skin, wound healing

## Abstract

Many biological systems have evolved circadian rhythms based on the daily cycles of daylight and darkness on Earth. Such rhythms are synchronised or entrained to 24-h cycles, predominantly by light, and disruption of the normal circadian rhythms has been linked to elevation of multiple health risks. The skin serves as a protective barrier to prevent microbial infection and maintain homoeostasis of the underlying tissue and the whole organism. However, in chronic non-healing wounds such as diabetic foot ulcers (DFUs), pressure sores, venous and arterial ulcers, a variety of factors conspire to prevent wound repair. On the other hand, keloids and hypertrophic scars arise from overactive repair mechanisms that fail to cease in a timely fashion, leading to excessive production of extracellular matrix (ECM) components such as such as collagen. Recent years have seen huge increases in our understanding of the functions of microRNAs (miRNAs) in wound repair. Concomitantly, there has been growing recognition of miRNA roles in circadian processes, either as regulators or targets of clock activity or direct responders to external circadian stimuli. In addition, miRNAs are now known to function as intercellular signalling mediators through extracellular vesicles (EVs). In this review, we explore the intersection of mechanisms by which circadian and miRNA responses interact with each other in relation to wound repair in the skin, using keratinocytes, macrophages and fibroblasts as exemplars. We highlight areas for further investigation to support the development of translational insights to support circadian medicine in the context of these cells.

## Introduction

Chronic, non-healing wounds have emerged as a major public health crisis associated with enormous negative impact on quality of life and healthcare budgets [1–3]. Factors driving the elevated incidence of chronic wounds include the ageing population, the diabetes epidemic, and the intersection of these phenomena, that is, diabetic patients living longer [1–4]. Wound management costs are dominated by diabetes-related amputations [2] and have been estimated at £5.3 billion annually [5] in the U.K. and a staggering $28.1–96.8 billion per year in the U.S.A. [6]. Comparative cost analyses are less readily available for the Global South, but it is noteworthy that every three in four (79%) people with diabetes live in low- and middle-income countries [7]. Further, the prevalence of diabetic foot ulcers (DFUs) reported for Africa (7.2%) was higher than that of Europe (5.1%) or Asia (5.5%) but lower than the 13.0% prevalence in North America [4]. However, the management of DFUs in Africa is burdened by a high rate of mortality during hospitalisation [8], perhaps reflecting a paucity of healthcare resources compared with high-income countries.

**Figure 1:**
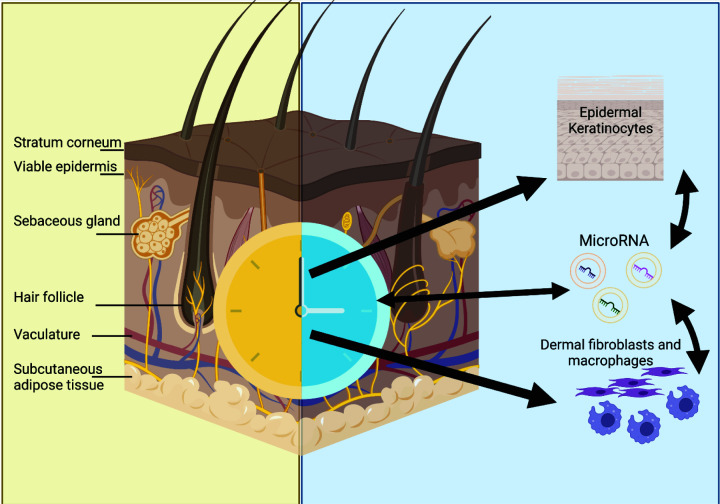
The normal physiology of the skin Multilayered organ containing stratum corneum or non-viable epidermis, the viable interfollicular epidermis consisting of keratinocytes, the vascular- and appendage-rich dermis and the subcutaneous adipose layer containing adipocytes. Biological clocks are known to regulate the behaviour of keratinocytes, fibroblasts and macrophages and may impact intercellular transfer of miRNAs via extracellular vesicles (EVs). Created in BioRender.

Healthy skin provides wrap-around protection for the body against the external environment, preventing mechanical and chemical damage to the underlying tissues and resisting microbial invasion [9]. The top layer of the skin is the epidermis, followed by the dermis and hypodermis (Figure 1). The epidermis consists primarily of keratinocytes that undergo terminal differentiation and, in combination with a lipid-rich matrix, form the epidermal barrier [10,11]. The dermis lies beneath the epidermis and contains fibroblasts that secrete extracellular matrix (ECM) proteins such as collagen, fibronectin and laminin and the hydrated proteoglycan gel in which these proteins are embedded [12]. The dermis also contains the microvasculature that supplies blood to the skin cells, as does the underlying hypodermis [13]. The hypodermis also contains subcutaneous adipose tissue (SAT) that supplies growth factors to the dermis and serves as an energy reservoir [14,15].

Wound healing is orchestrated by complex interactions among skin cells, immune cells and endothelial cells that underpin the various overlapping phases of wound repair, spanning haemostasis, inflammation, angiogenesis, proliferation and remodelling [15,16]. Reciprocal signalling between keratinocytes and fibroblasts [17], keratinocytes and immune cells such as neutrophils and macrophages [18] and between cells and the ECM [19,20] that provide robust systems to support wound healing.

While tissue repair is deficient in chronic non-healing wounds, it goes into overdrive in hypertrophic scars and keloids, which are fibroproliferative skin disorders associated with a pathological elevation of ECM components such as collagen, hyaluronan and fibronectin due to a massive increase in fibroblast numbers in the dermis [21–23]. Both keloids and hypertrophic scars are thought to arise from prolonged inflammation of the reticular dermis, with the magnitude of inflammation driving the growth of excessive scar tissue beyond the original wound borders in the case of keloids [21].

Over the last decade, it has become clear that small non-coding RNA molecules of the microRNA (miRNA) family play pivotal roles in the regulation of cells and associated processes during wound repair [24–26]. In addition, keratinocytes, fibroblasts, neutrophils and macrophages all exhibit circadian rhythms, ubiquitous daily changes in behaviour and physiology that are driven by endogenous biological clocks [27–30]. However, the effects of miRNA on the circadian properties of keratinocytes, fibroblasts and macrophages have received little attention in relation to wound repair. Likewise, the impact of core clock gene activity on miRNA expression in these cells during wound healing has not been established.

In this review, we explore mechanisms by which circadian and miRNA responses interact with each other in relation to wound healing in the skin. We begin with brief outlines of circadian biology and miRNA, respectively, and also introduce small extracellular vesicles (EVs) (exosomes), which have gained prominence as mediators of intercellular miRNA function in wound repair. We then examine a number of scenarios in which miRNAs may contribute to circadian responses associated with wound healing, with a focus on keratinocytes and macrophages.

## Brief primer on circadian rhythms

Circadian rhythms are fundamental processes that pervade biology and are thought to offer a selective advantage by optimising physiological and behavioural activities to appropriate temporal niches [31]. The endogenous pacemakers that generate these rhythms are found in most cells throughout the body and are underpinned by a set of so-called ‘core clock genes’, the transcriptional/translational activities of which form positive and negative feedback loops that oscillate with a period of approximately 24 h [32].

**Figure 2:**
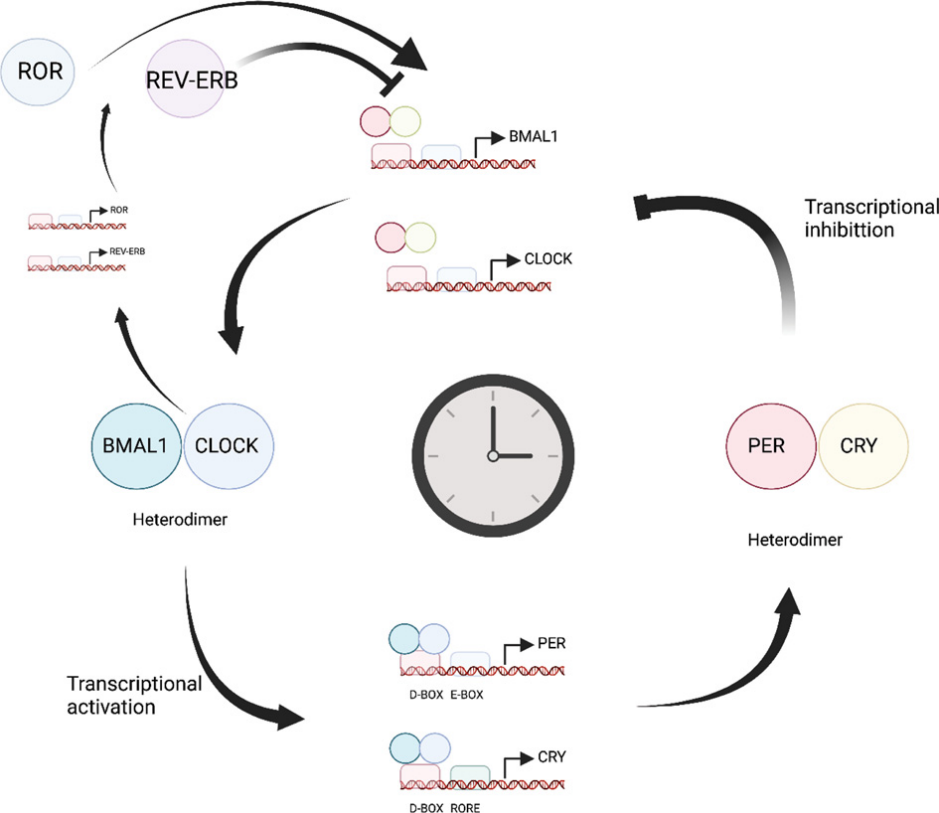
The core molecular clock The heterodimeric transcription factor BMAL1:CLOCK1 promotes the expression of PER and CRY proteins. The PER:CRY protein complex in turn represses BMAL1:CLOCK-dependent transcriptional activation of *PER* and *CRY* promotors. BMAL1/CLOCK also activates the expression of ROR and REV-ERB (NR1D1 and NR1D2) proteins. These confer additional control as ROR competitively activates BMAL1 expression and REV-ERB proteins competitively inhibit BMAL1 expression. Created in BioRender.

Central to this core molecular clockwork are the genes *Clock* and *Bmal1*, as well as the Cryptochrome 1/2 (*Cry1*/*2*) and Period 1/2 (*Per1*/*2*) genes. The positive drive for this mechanism is provided by a heterodimeric transcription factor consisting of BMAL1 (encoded by aryl hydrocarbon receptor nuclear translocator like, *ARNTL* and CLOCK (or its analogue neuronal PAS domain protein 2, NPAS2)) that acts on E-box elements in the promoters of the *Per* and *Cry* genes, activating their transcription (Figure 2). After translation, PER and CRY proteins form heterodimers that mediate a negative feedback limb by inhibiting BMAL1/CLOCK-dependent transcriptional activation of the *Cry1*/*2* and *Per1*/*2* promotors. BMAL1/CLOCK also activates the expression of *Rev-erb α/β* (also known as nuclear receptor subfamily 1, group D member 1, NR1D1 and NR1D2) and retinoic acid-related orphan receptors (*RORs α/β*). The REV-ERB α/β and RORs α/β proteins competitively inhibit and activate *Bmal1* expression, respectively. Various other genes and their proteins, including *Dbp* and *E4bp4*, and *Dec1*/*2*, form additional auxiliary loops. These proteins, together with dynamic phosphorylation and ubiquitination involving casein kinase 1δ/ε (CK1δ/ε) and F-box/LRR-repeat protein 3 (Fbxl3, a component of a ubiquitin ligase complex), provide further control over the system, fine-tuning the approximate 24-h rhythms that it generates [33]. In addition, post-transcriptional mechanisms spanning poly(A) tail length, RNA methylation, alternative splicing and miRNAs confer further regulatory control of the circadian clock, as reviewed very recently [34].

Rhythms driven by these cell-autonomous circadian oscillators must be synchronised with both the external and internal environments in order to generate biologically relevant timing signals, and this is achieved through the actions of a variety of factors, including environmental light, food, exercise and temperature [35]. In vertebrates, including humans, the extended circadian system consists of a ‘master’ circadian pacemaker located within the suprachiasmatic nucleus (SCN) of the hypothalamus and peripheral oscillators found throughout the rest of the body. A principal role of the SCN is to integrate both external time signals and feedback from the periphery, and transmit this information to peripheral oscillators, which use it to appropriately control the timing of local, tissue-specific, aspects of physiology [36].

## Circadian function in skin

Peripheral oscillators are found in almost all cells throughout the body and the skin is no exception. Both rhythmic activities and functional implications of circadian rhythms for the biology of skin have been widely described [37]. Daily variation in gene expression have been reported in the epidermis and dermis, as well as in cutaneous and SATs and dermal hair follicles [38–40]. At the cellular level epidermal keratinocytes, hair follicle cells, dermal fibroblasts and dermal macrophages all generate rhythms on a daily timescale, as do subcutaneous adipocytes [41].

Several mouse models have helped to establish key roles for core clock genes in the skin. For example, in *Bmal1*-knockout mice, SAT was depleted in aged mice compared with their younger counterparts [42]. Loss of SAT and other features of early ageing in the *Bmal1*-deficient mice were linked to circadian effects on reactive oxygen species (ROS) homoeostasis in selected tissues [42]. Indeed, we now know that deletion of *Bmal1* leads to the accumulation of ROS in both the epidermis and the dermis [43]. Further, adipose-derived stem cells and miRNA-loaded small extracellular vesicles (sEVs) released by these cells play key roles in wound healing, as reviewed elsewhere [44], though the impact of Bmal deletion on sEV density and composition in normal and wounded skin remains to be established. Defective wound healing is, however, seen in multiple mouse strains bearing core circadian clock gene mutations [45] and time-of-day of wounding differentially impacts on the rate of healing in diurnal and nocturnal species [46,47]. On the other hand, wound healing was accelerated in mice lacking NPAS2, and this was associated with enhanced collagen synthesis, migration, and proliferation of dermal fibroblasts [48].

Further studies have provided evidence for autonomous oscillations of molecular clock components in mouse skin, particularly in the epidermis and hair follicles, and rhythmic expression of PER2 was completely lost in Cry1/Cry2 double knockout mice lacking molecular clocks [49]. Crucially, Bmal1 controls the expression of stem cell regulatory genes and studies on mice lacking Bmal1 in basal epidermal keratinocytes, or on Per1/Per2 double knockout mice, revealed the molecular clock modulates stemness within the hair follicle stem cell niche [50]. Specifically, the hair follicle bulges of mice lacking Bmal1 had fewer proliferative cells and more dormant stem cells as the mice aged. On the other hand, a later independent study found that keratinocyte-specific deletion of Bmal1 results in constitutively elevated cell proliferation and abolishes time-of-day-dependent cell proliferation rhythms in the interfollicular epidermis and upper hair follicles [51]. Interestingly, this later study also found that accumulation of ROS in mouse skin appears to be temporally de-coupled from DNA replication, at least when the hair follicles of the skin were in the telogen (resting) phase [51].

Furthermore, the temporal segregation of biological processes has also been observed in human epidermal stem cells (EPSCs), with keratinocyte differentiation associated with late-night and early-morning hours, while DNA replication, UV protection and cell division proceed in the afternoon and evening hours [52]. Notably, studies on CLOCK (Clk/Clk) mouse mutants showed that even the hydration of the topmost layer of the epidermis, the stratum corneum, was regulated by clock genes [53]. This was associated with a rhythmic expression of aquaporin 3 that was observed in both mouse skin and in synchronised HaCaT keratinocytes [53].

Given that inflammation is a key early phase of wound repair, it is noteworthy that inflammatory responses, especially those driven by the Toll-like-receptor 7 agonist imiquimod (IMQ), have been linked to circadian dynamics in the epidermis [54,55]. Nakao and colleagues found psoriasis-like disease observed upon topical application of IMQ was attenuated or exacerbated in *Clock* and *Per2* mutant mice, respectively, compared with wildtype mice [54]. More recently, Andersen and colleagues showed that diurnal variation in both EPSC proliferation and epidermal thickness appeared to be lost upon treatment of mouse skin with IMQ [55]. Gene expression studies found that IMQ mobilised greater interferon (IFN) responses after daytime application to mouse skin and this was associated with greater daytime expression of approximately a quarter (53 out of 202) interferon-sensitive genes (ISGs) examined [55]. Importantly, IMQ dampened rhythmic Bmal1 expression and down-regulated *Rev-erbα (Nr1d1)* and *Dbp*. On the other hand, the inflammatory response to IMQ was potentiated in the epidermis of *Bmal1* knockout mice, with elevated expression of interferon-regulatory factor 7 (IRF7), a master regulator of ISG transcription, as well as increased serum levels of IFN-β [55]. Thus, *Bmal1* appears to support repression of inflammatory axes associated with ISG expression in the skin and type 1 IFN levels in the blood.

The above observations together point to the importance of core clock genes such as Bmal1 in skin processes associated with wound healing. Very recently, however, Reddy and colleagues presented evidence indicating that 24-h oscillations of the transcriptome, proteome, and phosphoproteome can persist in skin fibroblasts and liver slices from *Bmal1* knockout mice in the absence of exogenous stimuli [56]. Therefore, circadian gene expression in peripheral tissues might have mechanisms beyond *Bmal1* to provide oscillatory control of biological processes; a combination of E26 transformation-specific (ETS) family transcription factors and redox oscillations were uncovered as candidate drivers of such *Bmal1-*independent rhythmicity. However, these findings raise many questions with regard to BMAL1-independent ‘noncanonical’ circadian rhythmicity and have been challenged by independent investigators [57–60].

Intriguingly, in both nocturnal rodents and diurnal humans, wounds suffered during the daily active phase heal more rapidly than those suffered during the inactive phase [46], whilst exposure to dim light during the dark phase of a daily 24-h cycle, a stimulus known to be disruptive to normal circadian function, delays and impairs wound healing in mice [61]. At the gross tissue level, circadian control of skin physiology is taken to elevate mechanisms that minimise damage to the skin during the day, whilst promoting growth and repair mechanisms at night [37]. Importantly, an elegant recent study on mice found that while the majority of circadian gene expression in the epidermis may be associated with organismal factors linked to photic stimulation and the SCN, the core clock machinery of the epidermis appears to oscillate directly in response to cyclic changes in light, even though individual epidermal keratinocytes may have functioning cell autonomous clocks [62].

## MiRNAs at the interface of skin repair and rhythms

Since their discovery over two decades ago, miRNAs have been established as key regulators of normal and pathophysiological cell behaviour that repress gene expression in a post-transcriptional manner [63]. These small non-coding RNA molecules are generated from diverse genomic loci that yield primary miRNA transcripts which are processed to give rise to precursor then mature miRNAs that are typically ∼22 nucleotides long. The mature miRNA is loaded into Argonaute proteins to form the RNA-induced silencing complex (RISC) that executes the repression of messenger RNA transcripts through mechanisms involving mRNA degradation and, to a lesser extent, translation inhibition [64].

Recent years have seen a plethora of miRNAs implicated in wound repair processes spanning proliferation, resolution of inflammation, formation of granulation tissue, angiogenesis and migration [65–76]. Moreover, miRNA activity has been linked to circadian mechanisms in a wide variety of tissues, including the brain [77,78], heart [79], liver [80] and pineal gland [81]. Indeed, both rhythmic expression of miRNAs and miRNA alteration of circadian function have been described, as well as direct interactions between miRNAs and elements of the core circadian molecular machinery [77,82–84]. In skin, however, the relationships between miRNAs and circadian processes, including in cutaneous wound repair, remain poorly understood. This is especially the case in relation to autocrine and paracrine mechanisms of miRNA transmission between cells via EVs.

## EVs for intercellular miRNA transfer

One of the more recently discovered aspects of miRNA function is their role as mediators of intercellular communication, transferred from one cell to another via EVs. These EVs, which include sEVs known as exosomes (EXOs), microvesicles (MVs) and apoptotic bodies (ABs), convey diverse molecular cargoes to their target cells, including miRNA, RNA, DNA, proteins and metabolites. The miRNA cargo of EXOs appears to be distinct from that of ABs and MVs, which are more similar [85]. More importantly, EV-derived miRNAs from various mesenchymal and epithelial cells have been implicated in wound repair [86–96].

The three classes of EVs differ in size, but with some overlap: EXOs have a size range of 40–150 nm [97–99], while MVs range approximately from 100 to 350 nm [100], but can also reach approximately 1 μm [101]. ABs result from cellular breakdown during apoptosis and are the largest class, being 1–5 µm [101]. These overlapping size ranges present a challenge to the acquisition of pure populations of each class, as does overlapping epitope expression. Therefore, the International Society for Extracellular Vesicles (ISEV) [102] recommends providing quantitative details on isolation methods used and referring to all particles as ‘extracellular vesicles’, describing their physical features including size and epitope expression [102].

EXOs are derived from the endosomal pathway by invagination of the endosome membrane to form intraluminal vesicles within the multivesicular body (MVB). The formation of the MVB is regulated by the endosomal sorting complex required for transport (ESCRT), which recognises ubiquitinated membrane proteins and directs them into the intraluminal vesicles [103]. Subsequently, the MVB can either be subjected to the lysosomal degradation of vesicle contents or can fuse with the plasma membrane to release EXOs.

In contrast, MVs (also known as shedding vesicles, microparticles and ectosomes), which were first reported in platelets in 1967, are released by various immune cell types that circulate in the blood, including platelets and neutrophils [104–106], as well as tumour cells [107]. MVs are produced by budding directly from the plasma membrane rather than an intracellular precursor. Interestingly, studies are beginning show that the levels of specific MVs can vary in a circadian fashion [108,109].

Keratinocytes, fibroblasts and adipose tissue are rich sources of miRNA-loaded EVs [110–113]. Although the circadian behaviour of these cell is well-established, we do not know whether their ability to generate and release EVs follows a circadian pattern. Likewise, the impact of time of day on the loading of miRNA and other cargoes into EVs has not been defined. miRNA-loaded EVs from the above cell types target macrophages during wound repair [94,95], and macrophages also follow circadian rhythms [114]. With these perspectives in mind, we thus appraise keratinocytes and macrophages in relation to the interplay of miRNA and circadian processes with relevance to wound healing.

## Circadian miRNA possibilities in keratinocyte–macrophage interactions

Epidermal keratinocytes are critical for both normal skin physiology and successful wound closure and healing. Indeed, following physical damage to the skin, basal keratinocytes migrate to the wound site and proliferate, with daughter cells terminally differentiating to seal the wound. Keratinocyte migration and proliferation are controlled by an array of cytokines, growth factors and miRNAs, involving cross-talk among keratinocytes, dermal fibroblasts, activated immune cells and endothelial cells. The circadian clock has been shown to drive rhythmic expression of genes associated with epidermal keratinocyte physiology [115]. However, the relationships between such circadian behaviour and miRNAs have received limited attention, especially in relation to wound healing. In this regard, miR-21-5p is of particular interest because of its known ability to modulate wound repair, as we have reviewed elsewhere [25]. Further, diurnal expression of miR-21-5p has been observed in murine heart and lung, and the induction of miR-21-5p appeared to occur in a Per2-dependent manner [116]. In addition, exposure of mice or humans to intense light up-regulated miR-21-5p in murine hearts and human plasma, respectively [116]. Notably, the opsin family of light-sensitive G protein-coupled receptors associated with phototransduction, have recently been shown to be expressed in human epidermal keratinocytes and melanocytes [117–121]. Importantly, photic entrainment has been observed in mouse outer ear and macrovibrissal pad dermal tissues, with opsin 5 serving as a photopigment that drove phase-setting effects *ex vivo* (particularly over 370–415 nm) and photoentrainment of the local circadian clock *in vivo* [121]. Further, using a reporter assay for *per1* transcription, blue light (410 nm) has been shown to reduce *PER1* expression in normal human keratinocytes [122]. Together, these observations call for studies on relevant mouse models and *ex vivo* human skin to establish whether environmental light directs clock gene-dependent miRNA expression in skin cells, which may explain some of the observed effects of blue light on keratinocyte proliferation, differentiation and migration [118,123].

Very recent work has demonstrated circadian expression of miR-21-5p and miR-21-3p in atherosclerotic plaque macrophages, leading to diurnal changes in macrophage apoptosis [124]. Work is needed to determine whether wound macrophages can exhibit similar behaviour, especially given recent studies showing miR-21-5p from keratinocyte EXOs promoted the transdifferentiation of wound-resident macrophages into fibroblast-like cells [94,95]. This appears to be an important step in resolution of the inflammatory phase of wound healing and adds miR-21-5p to the growing number of miRNAs implicated in anti-inflammatory polarisation of macrophages [125,126]. However, whether miR-21 expression occurs in a circadian manner in cutaneous wound macrophages remains to be established.

**Figure 3:**
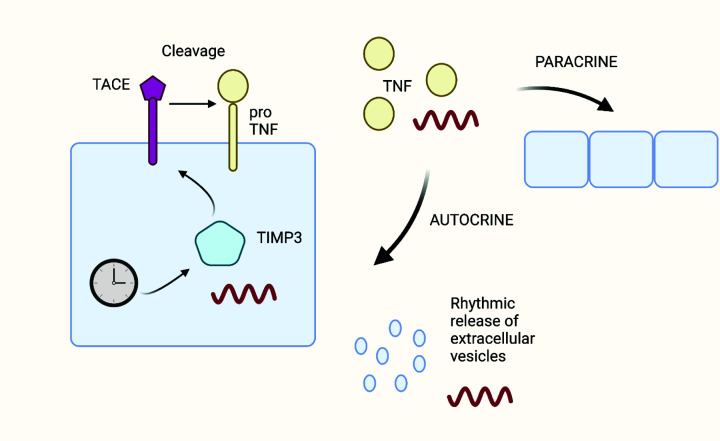
Hypothetical model for rhythmic release of keratinocyte EVs The core clock transcription/translation pathway regulates rhythmic expression of TIMP3, an enzyme required for synthesis of the disintegrin metalloprotease ADAM17 or TACE. TACE mediates extracellular cleavage of membrane-bound TNF to release the free growth factor. TNF can act in either an autocrine or paracrine fashion to regulate rhythmic release of EVs. Created in BioRender.

Interestingly, polarisation of the macrophages towards a fibroblast phenotype associated with resolution of inflammation was associated with miR-21-5p-dependent down-regulation of the transcription factor, Krüppel-like factor 5 (KLF5) and the phosphatase and tensin homologue (PTEN) [94]. KLF5 is a member of the Krüppel‐like factor family of transcriptional regulators and regulates diverse cellular processes, including proliferation, migration and differentiation [127]. PTEN dephosphorylates phosphatidylinositol (3,4,5)-trisphosphate to inactivate PI3K/AKT/mTOR signalling and is a well-established target of miR-21-5p [128]. At present, however, it is not clear whether secretion of keratinocyte EV or their contents occurs in a circadian fashion, nor do we know whether such putative circadian dynamics apply equally to EXO, MVs and ABs. However, we can hypothesise that EV release from keratinocytes occurs in a circadian fashion based on two observations. First, tumour necrosis factor α (TNFα), a major pro-inflammatory cytokine highly expressed at the wound site induces EV release from human keratinocytes [94]. Secondly, the secretion of TNFα is regulated by TIMP3, a CLOCK-controlled diurnal gene that inhibits the TNFα-converting enzyme (TACE; also known as ADAM17) responsible for release of membrane-bound TNFα into the surrounding milieu as active TNFα [129,130]. Ultraviolet radiation (UVB) down-regulated CLOCK, BMAL1 and TIMP3 mRNA and protein in human keratinocytes [129]. The expression on TIMP3 was periodic and the levels of TNFα and other inflammatory cytokines secreted under UVB conditions depended on TIMP3 expression. These observations suggest light-dependent diurnal variations in epidermal TNFα levels may generate rhythmic patterns of EV release from keratinocytes (Figure 3). Notably, TNFα and a plethora of other inflammatory markers were recently detected in suction blister fluid from skin [131], which may prove to be a useful biofluid for minimally invasive delineation of circadian variation in TNFα, EV and miRNA in human skin.

Full characterisation of keratinocyte EV-associated miRNA is important because apart from miR-21-5p, there may be additional exosomal miRNAs that target KLF5 and PTEN in wound macrophages. A search of TargetScan 7.2 (http://www.targetscan.org/vert_72/) revealed 20 conserved miRNA families that may regulate the KLF5 transcript, and 13 of these were among 381 keratinocyte miRNAs detected by Parker and colleagues in EVs from both primary human keratinocytes and the HaCaT keratinocyte cell line [132]; see Table 1. These studies relied on cultured cells and therefore need data from native or reconstituted human epidermal samples to support the key findings. Nonetheless, three miRNAs – miR-148-3p, miR-152-3p and miR-145-5p – had higher context scores than miR-21-5p, perhaps suggesting even more likelihood of KLF5 repression. This led us to examine the levels of miR-148-3p, miR-152-3p and miR-145-5p in EVs in the RNAseq data (GSE106453) reported by Parker and colleagues [132 which will become 85]. While miR-21-5p was relatively abundant (>12000 counts), amounts of miR-148-3p, miR-152-3p and miR-145-5p were low, with mean read counts of 1299, 26 and 46, respectively (Table 1). Hence whether these miRNAs can be transferred from keratinocytes to macrophages in functionally significant amounts remains to be seen. Nonetheless, it is noteworthy that miR-145-5p represses KLF5 in cancer cell lines [133–135] and bronchial epithelial cells [136], while miR-152-3p has been reported to suppress KLF5 in RAW264.7 macrophages, B cells and cervical cancer cells [137–139].

**Table 1:** MiRNAs predicted to regulate KLF5 and present in keratinocyte EVs

miRNA families broadly conserved among vertebrates	Cumulative weighted context score in TargetScan 7.2	Mean read counts in GSE106453 primary keratinocyte EVs	Predicted to regulate PTEN
miR-148-3p; 152-3p	−0.48	1299; 26	Yes
miR-145-5p	−0.41	46	Yes
miR-21-5p; 590-5p	−0.39	12067	Yes
miR-140-3p.2	−0.26	144	Yes
miR-143-3p	−0.18	4832	No
miR-23-3p	−0.18	381	Yes
miR-375	−0.16	38	No
miR-141-3p; 200a-3p	−0.15	1889; 112	Yes
miR-101-3p.1	−0.15	84	Yes
miR-182-5p	−0.15	3597	Yes
miR-25-3p; 32-5p	−0.12	333; 3	Yes
miR-96-5p; 1271-5p	−0.12	36; 3	Yes
miR-142-5p	−0.06	7	Yes

**Figure 4:**
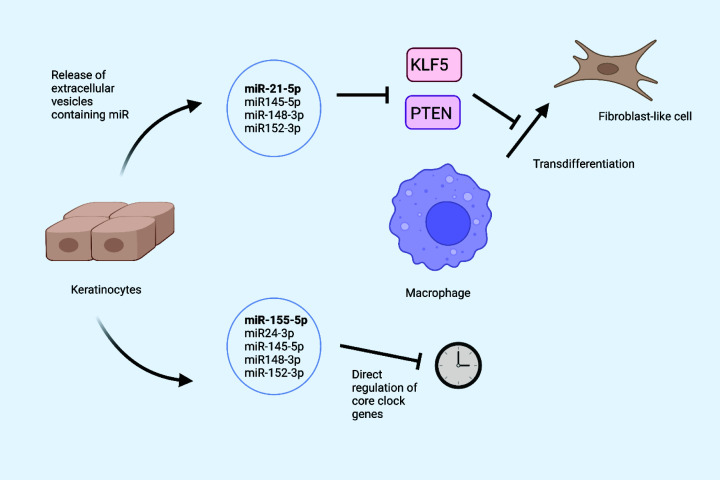
A model for regulation of macrophages by keratinocyte miRNA Epidermal keratinocytes release EVs replete with miRNAs [132]. Some of these miRNAs (miR-21-5p) promote polarisation (transdifferentiation to fibroblast-like cells) by repression of KLF5 and PTEN [94]. Others (miR-155-5p, miR-24-3p) modulate circadian rhythm [78,114]. A subset of miRNAs (miR-145-5p, miR148-3p, miR-152-3p) may target KLF5 and PTEN and also modulate circadian rhythm (see main text for details). Created in BioRender.

Notably, miR-145-5p and miR-148-3p were identified among 120 miRNAs reported very recently to modulate circadian periodicity in a genome-wide miRNA screen using an osteosarcoma cell line [140]. Hence, a picture emerges in which transfer of keratinocyte EV-derived miRNAs may concomitantly repress KLF5 and modulate the circadian properties of recipient macrophages (Figure 4). This raises questions as to whether the efficiency of miRNA-mediated macrophage polarisation depends on parallel changes in circadian parameters or, more broadly, the circadian proteome [141].

In addition, 11 of the 13 miRNAs predicted to target KLF5 were also predicted to target PTEN, including miR-21-5p, miR-148-3p, miR-152-3p and miR-145-5p (Table 1). There is evidence that miR-152-3p represses PTEN in human dermal fibroblasts (HDFs) and human umbilical vein endothelial cells [142,143]. Notably, conditional depletion of PTEN from mouse skin leads to nuclear accumulation of BMAL1 in the hair follicles and interfollicular epidermis [144,145]. This presumably exerts pro-healing effects as Squarize and colleagues very recently showed that depletion of BMAL1 slows epidermal regeneration in mouse skin by impairing keratinocyte proliferation [43]. Questions that arise include whether the effects of keratinocyte EV-derived miR-21-5p on macrophages are supported by other miRNAs such as miR-152-3p and whether these effects modulate the macrophage circadian behaviour through a PTEN:BMAL1 axis. It will thus be important, in the context of chronic diabetic wounds, to determine how diabetic factors (elevated inflammation, ROS, advanced glycation end products) affect the circadian features of the PTEN:BMAL1 pathway in keratinocytes, macrophages and other cell types such as fibroblasts, endothelial cells and neutrophils.

## Macrophages and miRNA: exosomal miR-24-3p as a putative regulator of PER2

Cross-talk between macrophages and keratinocytes is a crucial determinant of macrophage fate during tissue repair [94,95,146]. Epidermal keratinocytes release EXOs that are rich in miRNAs [147], approximately 400 of which are confidently annotated miRNAs [132]. Of the well-established miRNAs detected in keratinocyte EXOs, miR-24-3p is particularly interesting because using the PER::LUCIFERASE mouse, Takahashi and colleagues found that miR-24-3p repressed the PER2 transcript in mouse embryonic fibroblasts as well as various central and peripheral tissues, resulting in lower levels of PER2 protein [78]. This raises the possibility that elevation of miR-24-3p could directly repress PER2 in other cell types, such as macrophages recruited to the site of injury during wound repair, thereby regulating the circadian behaviour of these immune cells. Such elevation of miR-24-3p could be mediated via EXOs or other EVs engulfed by macrophages infiltrating the wound as there is evidence that macrophages recruited to the site of tissue injury selectively internalise keratinocyte-derived EXOs [94,95] and levels of miR-24-3p were very recently shown to be moderately elevated in serum and serum-derived EXOs of DFU patients [148].

Although the ability of miR-24-3p to target PER2 and circadian dynamics in macrophages has not been established, it is easy to envisage a scenario where miR-24-3p supports the suppression of PER2 amplitudes in response to TNFα, interferon γ (IFNγ) and Toll-like receptor (TLR) ligands Pam3CSK4 and lipopolysaccharide (LPS) [149]. Such repression of PER2 may drive changes in macrophage behaviour in relation to autonomous circadian metabolism and phagocytosis as reported very recently [141].

On the other hand, overexpression of miR-24-3p in macrophages has been shown to dampen secretion of pro-inflammatory cytokines, TNFα and IL-6 [150]. Given that the amplitudes of PER2 oscillations in macrophages were suppressed by TNFα [149], could miR-24-3p modulate circadian dynamics indirectly by depleting TNFα from the macrophage microenvironment during wound repair? In this scenario, exosomal miR-24-3p uptake would help decrease secreted TNFα levels, and this in turn would dampen PER2 rhythms. This calls for studies in which secreted TNFα levels in conditioned media of macrophages are compared directly with the amplitude of PER2 expression in the presence and absence of exogenous miR-24-3p. In addition, since cytokine (IL-4 and IL-13)-dependent polarisation of macrophages towards the anti-inflammatory M2 phenotype was dramatically enhanced in miR-24-loaded macrophages [150], it will be interesting to determine whether miR-24 controls PER2 rhythmicity under these conditions and how such putative modulation of PER2 affects macrophage polarisation.

Given that levels of miR-24-3p were very recently shown to be moderately elevated in serum and serum-derived EXOs of DFU patients [148], it is also worth investigating how serum-derived miR-24-3p may contribute to differential macrophage polarisation into distinct subsets spanning the M1 pro-inflammatory:M2 anti-inflammatory axis during wound healing observed in diabetic mice [151]. Experiments will need to be designed very carefully to determine the putative impact of serum-derived exosomal miR-24-3p on circadian dynamics in macrophages during wound repair and how this in turn modulates macrophage polarisation. However, the miRNA profiles of macrophages in normal versus diabetic skin wounds have not been defined to our knowledge.

### Macrophages and miR-155-5p-dependent regulation of BMAL1

The thrust of the above argument is that keratinocyte-derived miRNAs may impact the circadian biology of macrophages. This raises questions as to which other keratinocyte-derived miRNAs may be transferred to macrophages in a functionally relevant manner and how many of these may modulate circadian rhythms in the recipient macrophages. One strong candidate in this regard is miR-155-5p, which was also detected in keratinocyte EVs reported by Parker and colleagues [85]. In macrophages, miR-155-5p was one of the earliest miRNAs shown to be elevated in response to TLR stimulation [152]. Recent work from O’Neill and colleagues linked uncovered circadian variation in miR-155-5p expression, and linked this to the regulation of BMAL1 [114]. Induction of miR-155-5p and its host gene (*miR-155HG*) by LPS was two-fold higher in mouse peritoneal cells isolated at zeitgeber time 12 (ZT12) compared with ZT0. The elevated miR-155-5p expression was associated with increased susceptibility to LPS-induced sepsis and lethality and approximately three-fold increase in TNFα secretion. Analysis with TargetScan identified two miR-155-5p-binding sites in the 3′UTR of mouse *Bmal1* transcript and one miR-155-5p site in the human *BMAL1* mRNA, and the activity of the former was confirmed by luciferase reporter assays. Importantly, treatment of human macrophages with LPS reduced BMAL1 expression. This was associated with miR-155-5p as LPS-dependent depletion of BMAL1 was abrogated in human peripheral blood mononuclear cells (PBMCs) loaded with an miR-155-5p inhibitor [114]. Given that BMAL1 was known to attenuate NF-κB activation by sequestering CLOCK [153], the authors linked inhibition of miR-155-5p to reduced activation of NF-κB by LPS in PBMCs. What was less clear was the effects of the miR-155-5p:BMAL1 axis on the expression of canonical circadian genes with E-box responsive promoters. In any case, the picture that emerges is one where fluctuations in endogenous miR-155-5p in macrophages may be augmented by keratinocyte-derived miR-155-5p given that very recent work from Ghatak and colleagues showed that keratinocyte-derived EXO miRNA drives conversion of wound macrophages from proinflammatory into proresolution state [95]. Although they focused on miR-21-5p, other miRNAs from the 381 keratinocyte EXO miRNAs identified by Parker and colleagues may also be transferred from keratinocytes to macrophages via EVs, including miR-155-5p [132]. The overall dynamics of BMAL1 expression in macrophages would thus depend on the integration of endogenous and paracrine miRNAs (Figure 4). These ideas call for co-culture experiments of keratinocytes and macrophages, using keratinocytes in which *MIR155HG* has been deleted using gene-editing techniques.

## Do keratinocyte-derived EVs modulate the circadian clock in fibroblasts?

A recent study found high levels of miR-142-3p in EXOs derived from EPSC cell lines [96]. The EXOs enhanced wound healing in rat skin and appeared to dampen fibroblast differentiation to myofibroblasts by silencing transforming growth factor β (TGF-β1) signalling. Studies with a Transwell co-culture model with EPSC in the lower chamber and HDFs, in the insert allowed the authors to suggest EXO transfer from EPSCs to HDFs mediated silencing of TGF-β1 expression [96]. However, the roles of EXOs in this context need confirmation, for instance with experiments that incorporate inhibitors of EXO formation and release [154].

Nonetheless, it was interesting that the authors observed elevated EXO levels of two miRNAs that may target TGF-β1 signalling: miR-142-3p and miR-425-5p were approximately 30- and 40-fold higher, respectively, than those of the miR-16 control. This is interesting from a circadian perspective because miR-142-3p is a clock-controlled miRNA, expression of which is activated by BMAL1 [155]. In addition, miR-142-3p directly targets the 3′UTR of *BMAL1*, inhibiting BMAL1 expression [155]. The miR-142 gene was shown to contain an E-box to which CLOCK:BMAL1 bound. Functional studies confirmed CLOCK:BMAL1 co-expression increased activity of an *miR-142*-luciferase reporter and expression of miR-142-3p [155]. There is also evidence for miR-142-3p-dependent regulation of BMAL in HaCaT keratinocytes [156] and in the SCN of mice [157]. We thus have the intriguing possibility that the putative ability of exomosal miR-142-3p to inhibit fibroblast differentiation into myofibroblasts stems not only from suppression of TGF-β1 signalling but also from the miR-142-3p-dependent repression of BMAL1. Consistent with this, silencing of BMAL1 in normal human lung fibroblasts inhibited their differentiation into myofibroblasts [158]. More importantly, there is a clear need to establish a mouse model with epidermis-specific deletion of miR-142-3p to fully delineate the physiological relationships among miR-142-3p, BMAL1 and wound healing.

### Some miRNAs control the circadian clock and collagen expression

Very recent work from Kay and colleagues screened a library of 989 miRNA mimics against Bmal1 and Per2 luciferase reporters in a model cell line and identified 120 miRNAs that altered the circadian oscillations [140]. Surprisingly, neither miR-24 nor miR-155 (both discussed above) were reported among the miRNAs they identified, perhaps raising questions about the sensitivity of their assay. Notably, 54 of these miRNAs mapped to 35 miRNA clusters, including the miR-29b-1/miR-29a, miR-29c/miR29-b-2 cluster, the let-7a-1/let-7f-1/let-7d cluster and the miR-183; miR-96; miR-182 cluster. The miR-183/96/182 cluster was selected for further study given previous reports suggesting roles for this cluster in circadian processes [159,160]. The dominant mature miRNAs from the pre-miR-183/96/182 cluster are miR-182-5p, miR-96-5p and miR-183-5p. These mature miRNAs have seed regions that are similar but not identical and are predicted to target CLOCK and PER2. The circadian period lengths were altered in cells in which pre-miR-96, pre-miR-182 and pre-miR-183 genomic regions were deleted using clustered regularly interspaced short palindromic repeats (CRISPR)/CRISPR-associated protein 9 (Cas9) gene-editing approaches. Further, in mice lacking miR-183/96/182 expression, circadian patterns were altered at the behavioural level. Circadian parameters were also altered in the *ex vivo* cultures of the SCN, retina and lungs, those the precise changes differed. Reporter gene, miRNA and protein expression indicated that PER2 was a target of miR-96-5p as predicted, but the effects were all rather modest: for example, overexpression miR-96-5p dropped relative PER2 expression at the first time point measured from 0.7 to 0.5, and the putative effect of miR-96 deletion on PER2 expression in the knockout cells was not shown.

Notably, miR-29a-3p, miR-29b-2-5p and miR-29c-3p were also detected in the screen by Kay and colleagues for miRNAs that modulate circadian output [140]. One study has reported miR-29a/b/c-dependent regulation of human PER1 in human lung epithelial (A549) cells [161], providing a mechanistic basis for the effects of the miR-29 family on circadian rhythms. It is worth noting that depletion of miR-29 has been implicated in excessive collagen expression associated with fibrosis in diverse pathophysiological contexts, including keloids and hypertrophic scars [162–164].

## Circadian rhythmicity in collagen biosynthesis

Kadler and colleagues demonstrated very recently that mouse tendon collagen is controlled by rhythmic expression of four proteins, namely SEC61, TANGO1, PDE4D and VPS33B [165]. Rhythmic expression of these genes appears to separate production of procollagen at night from assembly of fibrils during the day. Cathepsin K (CTSK) is responsible for the rhythmic breakdown/degradation of collagen which overall leads to a maintenance of a homoeostatic level of functioning collagen within the tendon. Given the similarities between tendon and dermal fibroblasts [166], the physiological strains and biomechanical tensions that are in place on skin tissue when in the active period could position rhythmic collagen production as an additional factor in the circadian dynamics of fibroblasts during wound healing, alongside the rhythmic actin dynamics [46]. Interestingly, as PDE4D is a cAMP-specific phosphodiesterease, there may also be a role for circadian patterning of cAMP levels coupled to PDE4D expression. Indeed, Hastings and colleagues defined cAMP as an integral component of the circadian pacemaker in the SCN over a decade ago, and alterations in cAMP dynamics changed circadian parameters of the SCN as well as peripheral tissues and cultured fibroblasts [167].

From miRNA perspective, it is noteworthy that the expression of PDE4D may be controlled by several unrelated miRNAs, including miR-101-3p and miR-144-3p, miR-139-5p, miR-208a-3p, miR-203a-3p, miR-130a-3p and miR-219a-5p [168–173]. In contrast, there is limited evidence for miRNA regulation of *MIA3* (the gene that encodes TANGO1) apart from miR-30a-5p and miR-222-3p in colorectal cancer cells [174,175], even though the roles of TANGO1 in collagen secretion have received much attention recently [176–182]. Likewise, *VPS33B* and *SEC61A1* have not been established as miRNA targets, except for miR-218-5p in the case of *SEC61A1* [183].

## Conclusion

Chronic non-healing wounds such as DFUs, pressure sores, venous and arterial ulcers present clinical problems which require safe and effective treatment. The use of miRNA is an attractive and exciting approach for developing gene therapy for non-healing wounds. Recently, there has been increasing interest in the role of interaction of miRNA with circadian processes on wound repair. Moreover, miRNAs are known to function as intercellular signalling mediators through EVs. These are unique properties for miRNAs and indicate their great clinical potential in wound healing and repair. However, there is some controversy over the general ability of EVs to accumulate and deliver functionally relevant amounts of miRNAs to target cells [184]. Nonetheless, within a given tissue context (i.e. skin) paracrine EV-mediated miRNA transfer may be physiologically significant. Indeed, a very recent study found that miRNA sorting into EVs occurred in a cell-specific manner and sequence motifs, typically towards the 3′ end of mature miRNA (away from the seed region), regulate EV-loading versus cellular retention [185]. In any case, it is clear that miRNA expression through the delivery of mimics or inhibitors can enhance wound healing processes [25,26]. Further, with recent single-cell RNA sequencing uncovering four fibroblast subpopulations in normal scars and keloids, it will be interesting to determine miRNA associations with CRY1 and other core clock gene transcripts within each fibroblast subpopulation [186].

The literature reveals a wide range of miRNA that have been involved in wound healing processes. But the delivery of miRNA to wound tissues and its cellular uptake by wound cells are associated with many limitations related to the biological barriers and physicochemical nature of the molecules. A delivery system must be designed to overcome these challenges and ensure successful targeting of miRNA to the site of action without casing immune reactions. Advanced delivery strategies based on nanotechnology can provide the answers and solutions for the delivery issues. The future trend of innovative nano-miRNA therapy opens new avenues for advanced treatments that may lead to more effective, safer, faster wound healing and scarless skin.

## Open Access

Open access for this article was enabled by the participation of Liverpool John Moores University in an all-inclusive *Read & Publish* agreement with Portland Press and the Biochemical Society under a transformative agreement with JISC.

## Abbreviations

AB: apoptotic body
CRISPR: clustered regularly interspaced short palindromic repeats
Cry1/2: cryptochrome 1/2
DFU: diabetic foot ulcer
ECM: extracellular matrix
EPSC: epidermal stem cell
EV: extracellular vesicle
EXO: exosome
HDF: human dermal fibroblast
IFN: interferon
IMQ: imiquimod
ISG: interferon-sensitive gene
KLF5: Krüppel-like factor 5
LPS: lipopolysaccharide
miRNA: microRNA
MV: microvesicle
MVB: multivesicular body
NPAS2: neuronal PAS domain protein 2
PBMC: peripheral blood mononuclear cell
Per1/2: period 1/2
PTEN: phosphatase and tensin homologue
ROR: retinoic acid-related orphan receptor
ROS: reactive oxygen species
SAT: subcutaneous adipose tissue
SCN: suprachiasmatic nucleus
sEV: small extracellular vesicle
TGF-β1: transforming growth factor β
TLR: Toll-like receptor
TNFα: tumour necrosis factor α
